# Paramedian Frontal Flap Reconstruction for Nasal Defect Following an Accidental Amputation

**DOI:** 10.7759/cureus.61167

**Published:** 2024-05-27

**Authors:** Flávia Pereira, Sara Martins, Mariana Cebotari, Lígia Coelho

**Affiliations:** 1 Maxillofacial Surgery Department, Centro Hospitalar Universitário de São João, Porto, PRT

**Keywords:** maxillo-facial trauma, nasal amputation, nasal reconstruction, paramedian frontal flap, facial reconstructive surgery

## Abstract

This case report aims to present the successful reconstruction of a nasal defect in a 56-year-old male patient who suffered a partial nasal amputation due to a domestic accident involving a grinding wheel. The reconstruction was carried out using a paramedian frontal flap in a two-stage surgical process. Initially, the flap was designed and customized to match the dimensions of the defect, with a pedicle width of approximately 1.5 cm vertically. The flap was elevated in a distal-to-proximal manner, starting with subcutaneous dissection and progressing to periosteal dissection proximally. Weekly dressing changes were made using fatty gauze and fusidic acid ointment. Four weeks postoperatively, the flap pedicle was divided, and the brow was repositioned.

At the six-month follow-up, the patient showed satisfactory clinical outcomes with no functional complaints and was very pleased with the aesthetic result. Paramedian frontal flap reconstruction is a dependable technique for addressing nasal defects following traumatic amputation, providing favorable functional and aesthetic results. This case highlights the importance of careful surgical planning and technique in achieving successful facial reconstruction.

## Introduction

Facial reconstructive surgery, especially in cases involving the reconstruction of the nose after amputation, often requires employing various surgical techniques like paramedian frontal flaps, pericranial flaps, and osteoplastic procedures to restore both appearance and function to the affected area. One widely used technique is the paramedian frontal flap, extensively applied in complex nasal reconstructions for its ability to match the texture and color of the original nose skin [1]. This flap, nourished by the supratrochlear artery, is structurally compatible with nasal skin, proving successful in restoring nasal form and function [2,3].

In cases of nasal reconstruction following tumor removal, the use of paramedian frontal skin flaps is recommended, particularly for nasal tip cancers with specific characteristics [4]. In nasal reconstruction, the cross-paramedian forehead flap is recognized as a valuable design for extensive nasal reconstruction, demonstrating its effectiveness in addressing significant nasal defects [5]. Vascular studies have confirmed the safety and clinical benefits of the paramedian forehead flap, highlighting its adaptable anatomical features as a viable option for nasal reconstruction [1].

The positive impact of facial aesthetics and reconstructive surgeries on patients' quality of life has been extensively documented, showing improvements in body image perception, self-esteem, and overall well-being [5]. By incorporating advancements in surgical techniques and understanding their impact on patients' quality of life, facial reconstructive surgery continues to progress, providing new opportunities for patients in need of complex facial reconstruction.

## Case presentation

**Figure 1:**
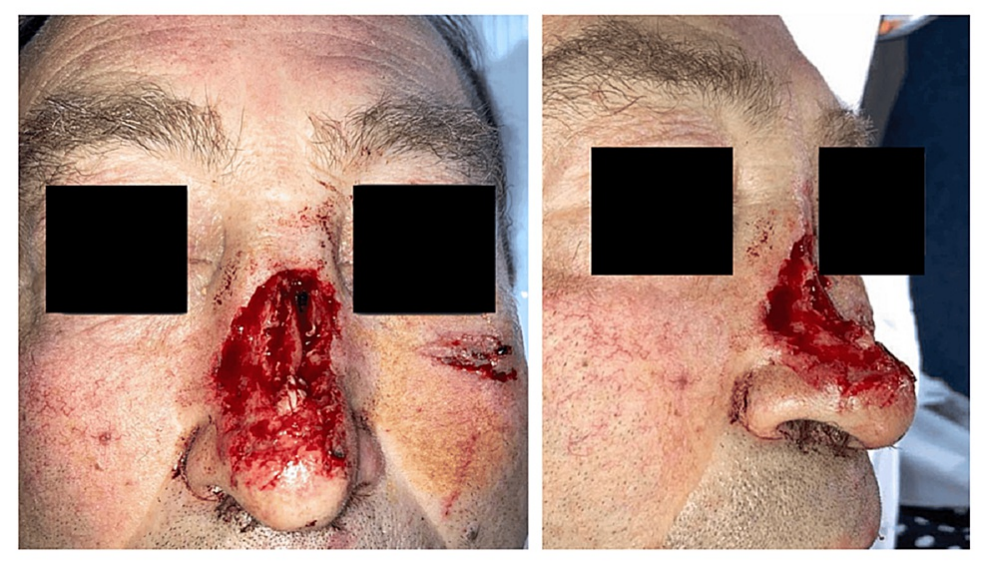
Initial images of the patient upon arrival at the emergency department

A 56-year-old male presented to the emergency room with a partial nasal defect resulting from a domestic accident involving a grinding wheel. Upon examination, a partial nasal amputation was noted, with a significant loss of tissue and disruption of the nasal contour (Figure 1).

**Figure 2:**
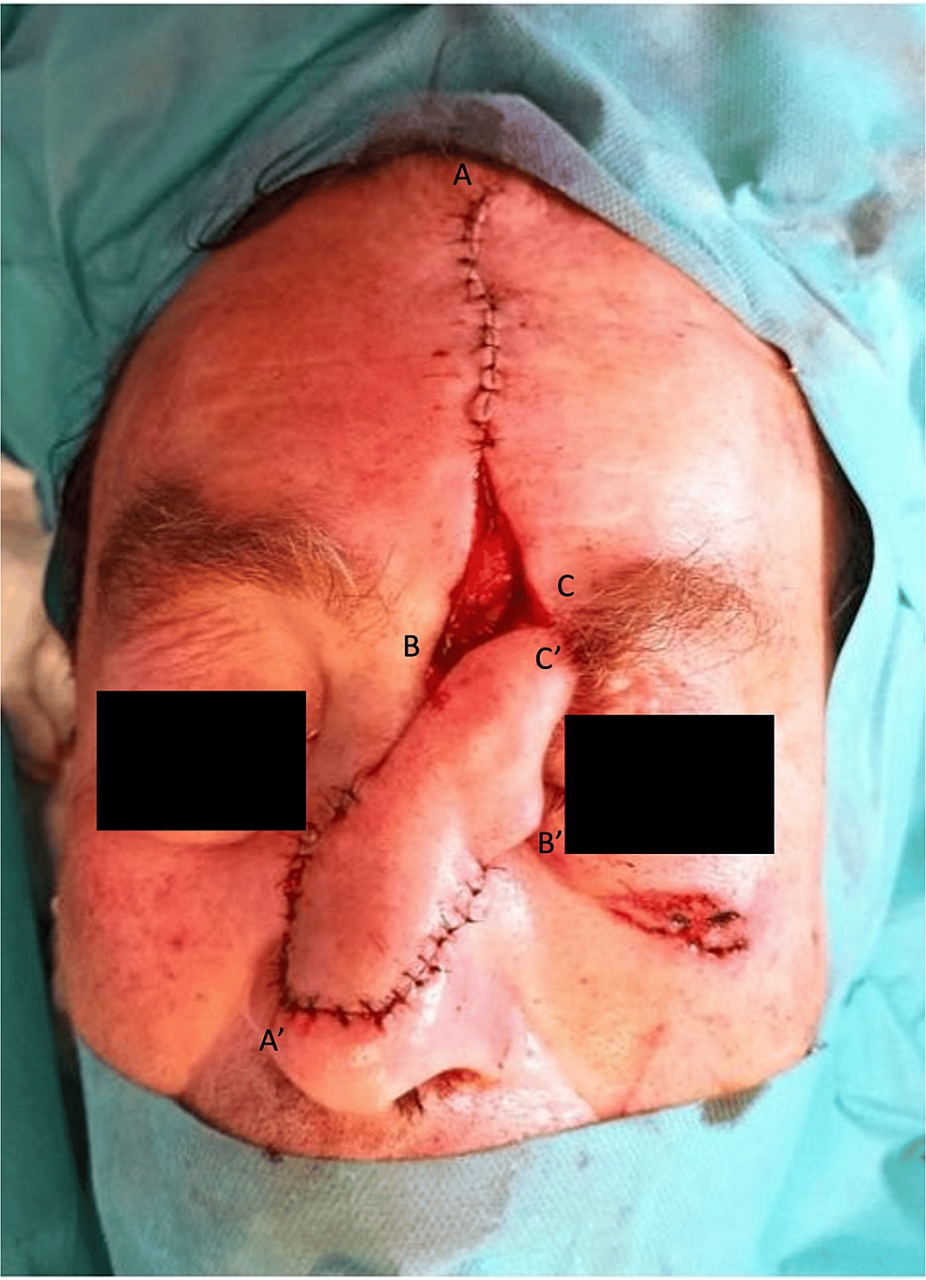
Stage 1 of the paramedian frontal flap procedure Immediate postoperative image. Letters (A, A', B, B', C, C') illustrate the counterclockwise rotation of the paramedian frontal flap and the positioning of the sutures during this stage.

Given the extent of the defect and the patient's desire for restoration, a decision was made to proceed with reconstruction using a paramedian frontal flap. The surgical procedure was performed in two stages to ensure optimal outcomes. In the 1st Stage, the paramedian frontal flap was meticulously designed to match the dimensions of the nasal defect. The flap, with a pedicle width of approximately 1.5 cm in the vertical direction, was raised in a distal-to-proximal manner, carefully dissecting through the subcutaneous and periosteal layers (Figure 2).

**Figure 3:**
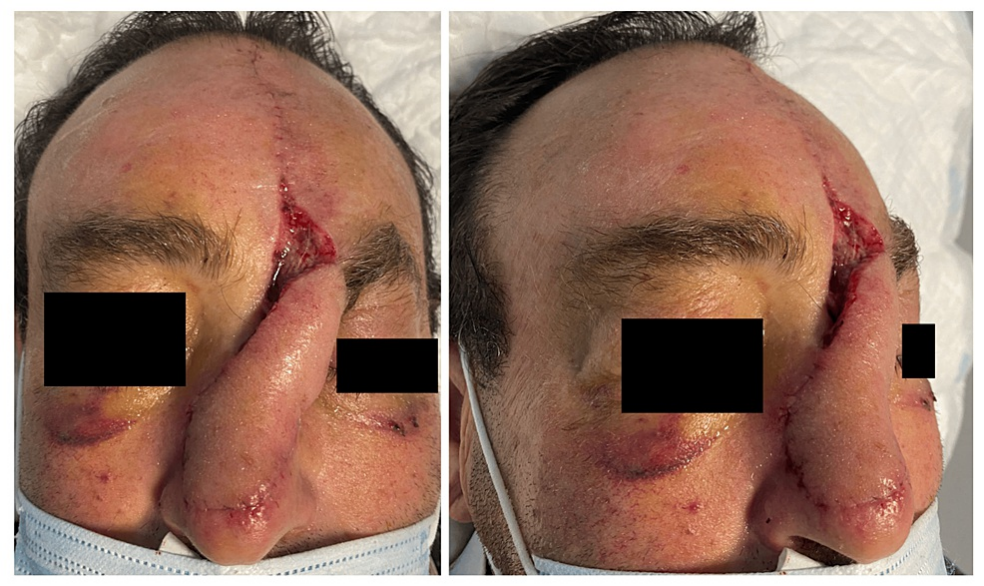
One week post-operation images, after suture removal

**Figure 4:**
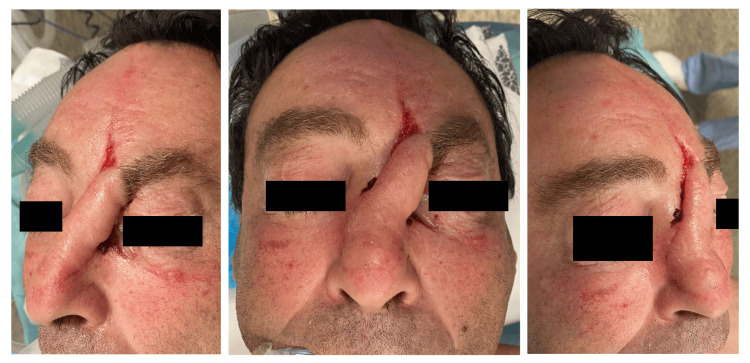
Four weeks post-operative images

Following the flap elevation, the patient underwent weekly dressing changes using fatty gauze and fusidic acid ointment to promote optimal healing and flap viability (Figures 3, 4)

**Figure 5:**
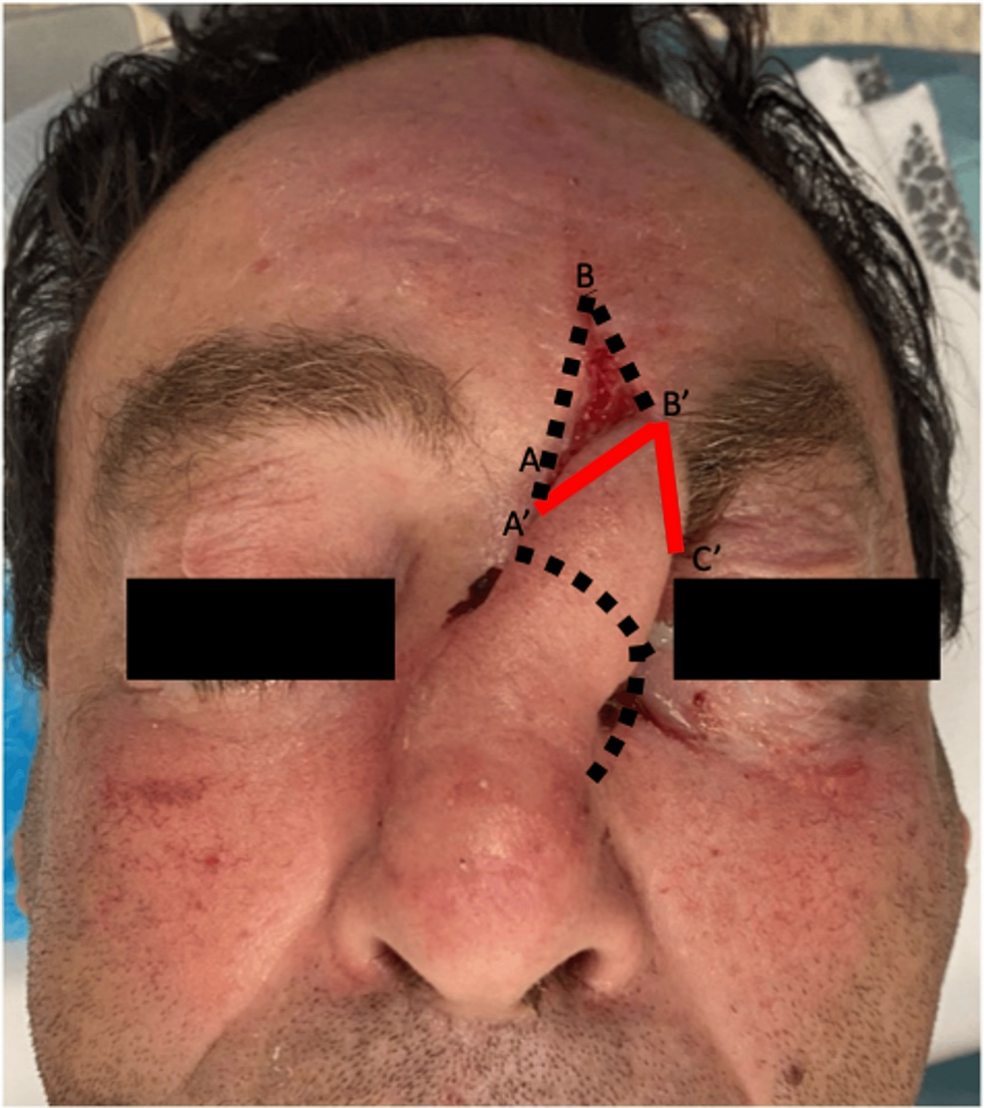
Surgical planning of Stage 2 procedure Pedicle sectioning with tissue repositioning for eyebrow elevation.

**Figure 6:**
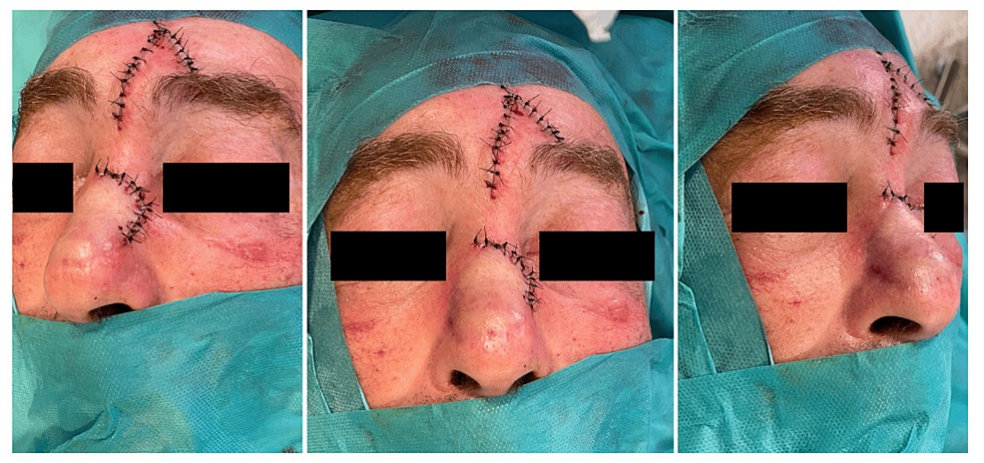
Stage 2 of the paramedian frontal flap procedure Immediate postoperative period.

After four weeks of meticulous postoperative care, the flap pedicle was deemed sufficiently vascularized, and the brow was repositioned in preparation for the final stage of reconstruction (Figures 5, 6)

**Figure 7:**
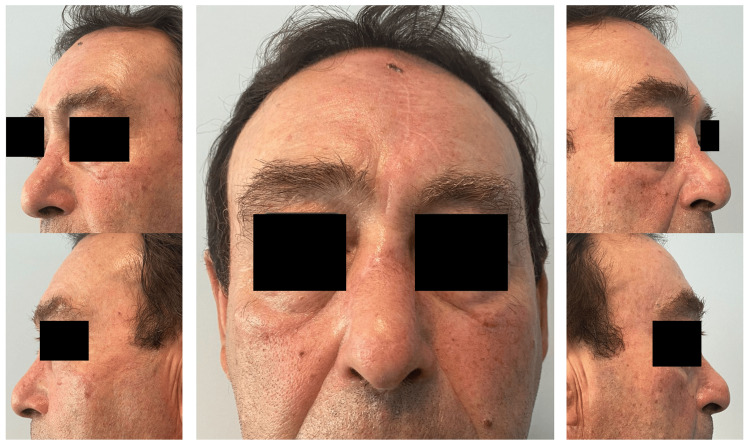
Results at six months post-operation

At the six-month follow-up evaluation, the patient exhibited remarkable clinical improvement with no functional complaints (Figure 7).

The reconstructed nasal contour harmoniously integrated with the surrounding facial features, restoring both function and aesthetics. The patient expressed high satisfaction with the outcome, emphasizing the significant impact of the reconstruction on his quality of life.

## Discussion

Injuries to the soft tissues of the midface, particularly the nose, are frequent reasons for seeking trauma care. Repairing these skin defects, especially on the nose due to its central facial position, is challenging and requires meticulous attention to both functional and aesthetic outcomes to ensure successful patient rehabilitation. Typically, soft tissue reconstruction in these cases involves the use of local and regional flaps. The specific approach depends on the defect’s location and size, the patient's age, and their preferences. Nasal skin, glabella, forehead, and nasolabial fold flaps are effective options for covering external defects, providing a good match in color and texture. Full-thickness skin grafts and healing by secondary intention are considered secondary options [6].

For nasal defect reconstruction, paramedian forehead flaps with a small pedicle are commonly utilized. The viability of these flaps depends on the presence of an arterial blood vessel within the pedicle. Therefore, it is crucial to understand the position and course of the forehead vessels when constructing the flap. The dorsal nasal artery, supratrochlear artery, and supraorbital artery, which are branches of the ophthalmic artery, provide essential blood supply to the forehead and interconnect with neighboring vessels. Additionally, these arteries form anastomoses with the frontal branch of the superficial temporal artery. Among these, the forehead branch of the dorsal nasal artery, though less well-known, is vital for supplying the central forehead. Alongside the supratrochlear artery, which runs along a paramedian line through the medial angle of the eye, it serves as the primary blood supply for the forehead flap. In cases of ophthalmic artery injury, the anastomosis between the angular artery and the dorsal nasal artery can be crucial for maintaining the flap’s blood supply [7].

Nasal reconstruction using the paramedian forehead flap generally involves a minimum of two surgical stages, with each operation typically spaced about three/four weeks apart. Some surgeons opt for a third stage to allow for additional refinement of the flap's contour. While harvesting the flap inevitably results in a vertical scar on the forehead, this scar is often not very noticeable. Overall, the cosmetic and functional results are usually very satisfactory for both patients and surgeons [8-10].

## Conclusions

In conclusion, the successful reconstruction of the nasal defect in this 56-year-old male patient highlights the efficacy of the paramedian frontal flap approach in addressing complex nasal deformities. Achieving great results usually requires a team that includes surgeons, a nursing team to assist with intermediate-stage dressings, and physical therapists, if necessary, to improve scarring. It is also important to mention that patients have a very important role in the recovery journey since they must be cooperative and willing to go through the process with all its intermediate stages, enduring some temporary discomfort. In this case, through meticulous surgical planning and postoperative care, optimal functional and aesthetic outcomes were accomplished. The dressing care was performed by the medical team, and physical therapy was not needed since the functional and aesthetic outcomes were very satisfactory, and the patient was happy with the results.
